# Sport-based interventions and health in prisons: The impact of Twinning Project on prisoner wellbeing and attitudes

**DOI:** 10.1177/13591053241272188

**Published:** 2024-08-20

**Authors:** Linus Peitz, Martha Newson

**Affiliations:** 1 University of Greenwich, UK; 2 University of Kent, UK; 3 University of Oxford, UK

**Keywords:** prisoner health, social cure, social identities, sport-based intervention, Twinning Project, wellbeing

## Abstract

Social isolation and lack of support networks are key factors contributing to mental health problems among incarcerated people, which, in turn, are associated with an increased risk of reoffending. Enabling prisoners to form positive group relations and social identities is one approach to address the cycle of ill health and incarceration. We examine the impact of a football-based intervention, the Twinning Project, on prisoners’ wellbeing and social relations. Longitudinal and correlational analyses of data from *N* = 164 UK prisoners show how social bonding is linked with significant boosts to psychological need satisfaction, life satisfaction, efficacy beliefs as well as higher levels of wellbeing.

## Introduction

Incarceration has long been associated with physical and mental ill health (Fazel et al., 2016). Data from UK prisons showed that the rate of attempted suicide among males was 3.9 times higher compared to the general population (ONS, 2023), coinciding with a higher prevalence of other ill health indicators, such as anxiety disorders and depression (NICE, 2017). Poor mental wellbeing has, in turn, been linked to higher recidivism likelihood (Wallace and Wang, 2020). This emphasises the importance of improving prisoner health, both with regard to individual wellbeing and the broader societal economic and social costs of reoffending (e.g. £18.1bn per annum in the UK, Newton et al., 2019).

### Sport-based interventions

One promising approach with the capacity to address both health and criminal behaviour outcomes is the application of sport-based interventions. Sports programmes are a popular tool to address various societal issues, providing an attractive platform and accessible infrastructure to promote programmes related to education, health and social justice (Kingett et al., 2017; Schulenkorf et al., 2016). Within the context of criminal justice, sports programmes have been utilised both in prevention and intervention designs. For example, participation in yoga courses during incarceration was found to be associated with lower 5-year reoffending rates (Kovalsky et al., 2020), as well as with improved wellbeing (higher positive affect and lower stress) among prisoners (Bilderbeck et al., 2013). Programmes based on team sports have also been effectively deployed, such as British rugby interventions that were linked with lower recidivism (Meek, 2012) and improved attitudes towards aggression and crime (Williams et al., 2015). A recent meta-analysis by Jugl et al. (2023) evaluated the overall impact of sport-based programmes, showing moderate effects on crime-related outcomes (including reoffence rates, self-reported behaviour and attitudes related to crime) and large effects on psychological outcomes (including self-esteem, stress-related burdens and depressive symptoms). However, more research is needed to fully understand the mechanisms by which these programmes reach their full potential, maximally benefiting participants and wider society.

### The social cure approach

One potential mechanism is described by the social cure theory, which posits that membership in social groups plays a critical role in shaping values, attitudes and behaviours (Haslam et al., 2018). A recent meta-analysis shows that interventions building social identification among a group positively impact participants’ health (Steffens et al., 2021). Indeed, the social cure approach has been successfully applied to improve wellbeing for prisoners for example, involved in a group-based dance programme (Kyprianides and Easterbrook, 2020), or in a social-support programme (Aureli et al., 2020). More broadly, research has demonstrated the potential impact of positive social identities for people involved in the justice system (Kjellstrand et al., 2022; Kyprianides et al., 2019).

### The present research

Here we analyse data from the Twinning Project, a nationwide football-based intervention that pairs major football clubs with their local prisons. The programme is designed to improve participants’ self-esteem and employability by delivering coaching and other accredited football qualifications in a group setting. The programme is aimed at prisoners within 12–24 months of release with implicit minimum levels of fitness and literacy, and people who have committed sexual offences or are on low *Incentive and Earned Privileges* (IEP) levels are not permitted to take part (see Supplemental Appendix A for additional details on the programme’s structure).

We test whether participation in the Twinning Project is associated with: (a) improvements to health and wellbeing; (b) improvements to life attitudes; and (c) stronger social bonds. In exploratory analyses, we further test the social cure hypothesis by examining the relationship between positive outcomes and improved social connections. Hypotheses about pre-post tests were pre-registered as part of a larger evaluation of the Twinning Project at https://osf.io/rqm87/?view_only=e314d8e1b1344e64bca11ffaee20fae7.^1^ The research was approved by the ethics board of the University of Oxford (SAME_C1A_19_016) and the National Research Committee (2019-215).

## Methods

### Sample

Data from adult prisoners was collected between March 2022 and May 2023 from 18 UK prisons where staff had received training to use the UPSHOT monitoring system (a list of institutions is provided in Supplemental Appendix B). Although the programme serves both male and female cohorts, no female cases were captured. The implications of this gender disparity are discussed. Data of *N* = 316 Twinning Project participants was available. Of the 316 cases, 152 were incomplete, either missing pre-treatment (*n* *=* 21) or post-treatment data (*n* = 131). Missing data was mainly clustered within specific institutions and to a lesser degree associated with participant baseline characteristics.^2^ The following analyses are based on complete cases only, and the implications of missing data patterns are discussed. The sample contained *N* = 164 participants (age groups: <25 = 18.9%, 25–29 = 17.7%, 30–39 = 39.6%, 40–49 = 12.8%, >50 = 3.6%, unknown = 1.8%). The sample was majority white (72.6%, Asian = 3.7%, Black = 7.9%, Mixed = 9.8%, Other = 1.2%, Prefer not to say = 4.9%). The study was adequately powered to identify small effects at 0.05 error probability (minimum sample required *N* = 156 for paired sample *t*-tests, according to power calculations via G*power (Faul et al., 2007)).

### Procedure

Twinning Project participants completed paper copies of a survey designed by HMPS at the first and last session of the programme. These were manually transcribed (i.e. digitised) by the corresponding prison officer into an online databank, reflecting the restrictions around digital technology for people in prison.

### Materials

A full list of items, scales and internal reliability scores is provided in Supplemental Appendix D. The survey was designed by HMPS, and two measures of social relations (identity fusion and social identification) were added by the research team.

Health. Indicators of health included a measure of general physical health and a measure of physical activity frequency (days in the past week).

Wellbeing. The survey also included two items on emotional states (anxiety and happiness). Six items from the Warwick-Edinburgh Mental Wellbeing scale (WEMBS; Tennant et al., 2007) captured the satisfaction of important psychological needs including relatedness, purpose, resilience and control.

Life attitudes. Life satisfaction was measured with two items. Future optimism and personal efficacy were measured with one item respectively. Desirable custodial attitudes (e.g. motivation to work on offending behaviour) were measured with three items.

Social relations. The survey contained multiple measures of group attachment and prison relationships. Identification with *the Twinning Project* and with *other criminals* was measured with Postmes et al.’s (2013) single-item scale (‘I identify with *[target group]*’). Identity fusion with these two groups was measured using a pictorial measure (based on Swann et al., 2012), where participants indicated their feeling of closeness between themselves and the target group by selecting one of five pictures showing two circles ranging from *no relationship* to *total oneness* with a group. Respondents also indicated whether they had good relations with prison officers and other prisoners.

## Results

### Pre-post tests

We conducted paired sample *t-*tests to compare levels of wellbeing, life attitudes and social relations between the start and the end of the programme. An initial observation of the data distribution showed that baseline levels of almost all^3^ measures were significantly skewed towards the favourable end of the scales (i.e. ratio of skewness/standard error of skewness >1.96), pointing to ceiling effects among the available sample.

Nevertheless, participants showed significantly higher levels of psychological need satisfaction after the programme (*t*(163) = −2.93, *p* = 0.004, *d* = 0.23), as well as higher levels of life satisfaction (*t*(163) = −5.31, *p* < 0.001, *d* = 0.42) and self-efficacy beliefs (*t*(161) = −2.71, *p* = 0.007, *d* = 0.21; see Supplemental Appendix C for full results). Importantly, identification with the programme also increased significantly (*t*(140) = −2.93, *p* = 0.004, *d* = 0.25), whereas identification with other criminals did not change (*p* = 0.397). Levels of identity fusion, a more intense form of social bonding, remained stable, as did relations with POs and other prisoners, as well as custodial attitudes^4^ (*p*’s > 0.146).

### Correlations between wellbeing and social relations

Zero-order correlations between difference scores of variables with significant pre-post changes showed that those who identified more strongly with the Twinning Project also experienced significantly increased need satisfaction (*r* = 0.19, *p* = 0.024), but there was no direct association with changes in life satisfaction (*r* = 0.16, *p* = 0.065), efficacy beliefs (*r* = 0.09, *p* = 0.288) or custodial attitudes (*r* = 0.16, *p* = 0.062). Changes in psychological need satisfaction correlated significantly with increased life satisfaction (*r* = 0.40, *p* < 0.001) and efficacy beliefs (*r* = 0.22, *p* = 0.005).

We used Hayes’ (2022) Process macro for SPSS (model 4) to test for potential indirect effects of identity change on life attitude change (i.e. efficacy beliefs, life satisfaction) via increased levels of psychological need satisfaction, while controlling for demographic factors (age, ethnic minority status, disability status). We found significant indirect effects of identity change via psychological need satisfaction change for the models predicting change in life satisfaction (*B* = 0.06, SE = 0.03, 95% CI = 0.008–0.273), and change in personal efficacy (*B* = 0.03, SE = 0.02, 95% CI = 0.001–0.084). Participants who reported a disability also experienced stronger improvements to life satisfaction (*B* = 1.12, SE = 0.46, 95% CI = 0.216–2.023). We tested the same mediation model to predict desirable post-treatment outcomes which had not changed over time, adding pre-treatment levels as additional covariates. We found further indirect effects of identification change on post-treatment levels of future optimism (*B* = 0.06, SE = 0.03, 95% CI = 0.014–0.132), happiness (*B* = 0.09, SE = 0.07, 95% CI = 0.004–0.250) and anxiety (*B* = −0.12, SE = 0.07, 95% CI = −0.248 to −0.002; see Supplemental Appendix C for full results).

## Discussion

Our analyses suggest that participation in the Twinning Project has a positive impact on participants’ wellbeing. Specifically, the pre-post analyses showed significant improvements to efficacy beliefs and life satisfaction, as well as increased identification with the programme and increased levels of psychological need satisfaction. We also found evidence for the social cure hypothesis, such that feelings of closeness to the Twining Project were indirectly associated with improvements to life satisfaction, personal efficacy and higher levels of future optimism and happiness after the programme ended, via increased psychological need satisfaction.

Compared to other group-based interventions in prison, the Twinning Project offers a highly desirable hook: football. With over 3.5 billion fans globally (WPR, 2023) and lifelong loyalty associated with football identities (Newson et al., 2016), social bonds to the Twinning Project were already high at baseline. We see the Twinning Project as an opportunity for groups of participants who are stigmatised or otherwise excluded from mainstream curricula to participate in a meaningful way. These are groups not actively encouraged to participate, such as those with a history of trauma, with experience of the care leaver system or with poor behaviour in prison. We recommend extending recruitment to see more significant improvements.

The present research has some limitations. First and foremost is the lack of a suitable control group, which, in combination with the selection criteria of the programme (aimed towards healthy and well-behaved prisoners), prohibits generalisations to the wider prison population. The lack of a follow-up period, and the inability to link data across sources, for example, to evaluate whether changes in wellbeing, life attitudes and social relations were also reflected in custodial behaviour, also limits the scope of the interpretation to immediate treatment effects. Our insights are also to be interpreted in the context of the data that was- and was not available. As such, we can only speak to the male population which has different needs with regard to personal health compared to female prisoners (Tyler et al., 2019), and thus it remains unclear if the Twinning Project has a comparable impact on its’ female cohorts. There was also the issue of missing data. Much of this was attributed to institutional differences, hinting at suboptimal data collection and transcription procedures. Incomplete responses were also, to a lesser extent, associated with baseline future optimism and bonding to the programme which may have skewed results to appear more favourably. Conversely, an evaluation of the Twining Project’s impact on prison behaviour (Newson et al., under review) suggests the programme was particularly impactful for participants who showed less desirable baseline behaviours.

The data presented here add to existing evidence that the Twinning Project indeed leads to positive outcomes for participants (Newson et al., 2024), and further deepens our understanding of how these positive outcomes are achieved. Triangulating previous findings, we found that participants became increasingly bonded with the programme over time, which was associated with greater satisfaction of psychological needs and more positive outlooks on life and the future in general. In the context of the mental health crisis among people involved in the British justice system, our findings reiterate the importance of pro-social relations for prisoners and highlight the capacity of group-based programmes to impact wellbeing, even if their focus is educational or vocational. In line with other recent contributions to the social-cure approach in criminal justice settings (Kyprianides and Easterbrook, 2020), programme providers should consider how programme designs can enable participants to benefit from social bonding mechanisms in this challenging environment. The next step however will be to support formerly incarcerated people as they leave the prison gates to sustain meaningful and positive social relationships – can the ‘football cure’ be maintained after release?

## Supplemental Material

sj-docx-1-hpq-10.1177_13591053241272188 – Supplemental material for Sport-based interventions and health in prisons: The impact of Twinning Project on prisoner wellbeing and attitudes Supplemental material, sj-docx-1-hpq-10.1177_13591053241272188 for Sport-based interventions and health in prisons: The impact of Twinning Project on prisoner wellbeing and attitudes by Linus Peitz and Martha Newson in Journal of Health Psychology

## Notes

1. We deviated from the pre-registered analyses plan by conducting paired sample *t*-tests instead of ANCOVAs with a paired (repeated measures) factor because we did not obtain an indicator of *treatment duration*, which would have been the intended covariate. Furthermore, the pre-registered control group analyses were deemed inappropriate due to significant differences between the treatment and comparison groups with regard to demographic characteristics and baseline levels of health and wellbeing (see Supplemental Appendix B1).

2. Logistic regression analysis showed that the majority of variance in complete versus incomplete responses was explained by the institutional source of the data (χ^2^(17) = 165.99, *p* < 0.001. Nagelkerke *R*^2^ = 59.9%), while baseline differences in fusion to the Twinning Project and future optimism accounted for a smaller, but significant share of the variance (Δχ^2^(7) = 15.23, *p* = 0.033, Nagelkerke *R*^2^ = 63.8%). Full results of this analysis, as well as breakdowns of missing data by individual institutions and variables, can be found in Supplemental Appendix B.

3. Except for the following measures: Life satisfaction (two items, Skewness ratio = 1.17), Fusion to the Twinning Project (Skewness ratio = 0.51).

4. The initial pre-post test for custodial attitudes showed a significant reduction (*t*(163) = 2.69, *p* = 0.004). A subsequent one-way ANOVA examining custodial attitude change between prisons (*F*(10, 153) = 4.59, *p* = 0.002) showed that the significant effect was due to change in one institution (Δ*M* = −2.09) more than seven times the sample average (Δ*M* = −0.28). Cases from this institution did not differ from others on any other metric (*p*’s > 0.117), nor did differences between any of the other institutions (*p*’s > 0.086). Thus, we interpreted the deviation in custodial attitude as an outlier, plausibly caused by a transcription error, and we report pre-post tests for this outcome excluding the respective cases.
