# Supplementary material for: Sport-based interventions and health in prisons: The impact of Twinning Project on prisoner wellbeing and attitudes
Source: J Health Psychol. 2024 Aug 20;30(6):1408–14. doi: 10.1177/13591053241272188 (PMC12053259; doi:10.1177/13591053241272188)
Supplement: sj-docx-1-hpq-10.1177_13591053241272188 – Supplemental material for Sport-based interventions and health in prisons: The impact of Twinning Project on prisoner wellbeing and attitudes [file sj-docx-1-hpq-10.1177_13591053241272188.docx]

APPENDIX A: Information about the Twinning Project (based on the Template for Intervention Description and Replication, TiDierR, https://www.equator-network.org/reporting-guidelines/tidier/)

- 1. The Twinning Project (TP) is a sport-based intervention that pairs prisons with local professional sports teams (association football, rugby) to provide entry-level accredited coaching or refereeing courses to prisoners. The intervention is provided in adult-only male and female prisons in England and Wales, including all security categories. The goal of TP is to increase participants’ self-esteem and employability and thereby reduce reoffending rates after release, as well as to improve behaviour and wellbeing while incarcerated.
  2. Programmes are delivered face-to-face by teams consisting of at least one prison officer (typically a Physical Education Instructor) and one member of staff of the sports club (a coach) and can take between 1 - 12 weeks (minimum 5 sessions) to complete. Individual sessions are delivered face-to-face to groups of about 10-15 prisoners, using the facilities available in the respective prisons, which may or may not include outdoor facilities. The most common version of the programme provides an entry level football coaching qualification, which consists of both theoretical and practical sessions. Materials reflect the official FA ‘Introduction to coaching football’ course, but coaches have the ability to adjust materials according to cohort needs (e.g., shorten text on slides, bring additional material for illustration purposes).

Cohorts are self-selecting: participants apply to take part in the Twinning Project, which is advertised and tends to be well known in the prison gym and in other areas of the prison. Applications are assessed by prison and provider staff, who follow a number of selection guidelines. This includes a stipulation by the football clubs that prisoners with convictions for sexual offences are not permitted on the programme. Furthermore, the Twinning Project charity’s focus on employability has informed the guideline that participants should ideally have no more than 12 or a maximum of 24 months left to serve by the start of the programme. Moreover, the programme’s educational and physical components require an implicit degree of literacy and physical fitness. Additionally, the prison system has internal rules about programme participation wherein prisoners on lower Incentives and Earned Privileges (IEP) levels are not entitled to partake in specific programmes. There is usually a waiting list to take part in a programme.

APPENDIX B: Analyses of Comparison Group and Incomplete Response Data

| **Table B1**  *χ^2^ and Independent Sample T-Tests Comparing Demographic Characteristics and Baseline-Level Indicators of Wellbeing and Life Attitudes Between Twinning Project Participants and a Comparison Group* | | |
| --- | --- | --- |
|  | Twinning Project | Comparison Group |
|  | %/ M(SD) | %/ M(SD) |
| **Demographics** |  |  |
| Age |  |  |
| <25 | 22.6% | 13%* |
| 25-49 | 74.1% | 80% |
| >50 | 3.3% | 7% |
| Ethnicity |  |  |
| Asian | 4.2% | 10.9%** |
| Black | 9.6% | 21%** |
| Mixed | 8.3% | 15.1%* |
| White | 72.4% | 39.5%*** |
| Other | 1% | 11.8%*** |
| Prefer not to say | 4.5% | 1.7% |
| Disability | 21.2% | 2.5%*** |
| **Baseline Levels** |  |  |
| Physical health | 3.61 (0.96) | 3.52 (0.84) |
| Physical activity | 4.37 (1.95) | 4.30 (1.53) |
| Happiness | 7.34 (2.18) | 5.49 (2.47)*** |
| Anxiety | 2.36 (2.77) | 4.33 (2.56)*** |
| Psychological resources | 3.82 (0.72) | 3.35 (0.56)*** |
| Life satisfaction | 6.67 (1.99) | 5.67 (2.03)*** |
| Custodial attitudes | 4.32 (0.88) | 3.89 (0.75)*** |
| Relations with POs | 3.39 (0.94) | 3.64 (0.85)** |
| Relations with prisoners | 4.04 (0.83) | 3.64 (0.87)*** |
| *Note.* * *p*  < .05, ** *p* < .01, *** *p* < .001. Twinning Project N = 295, Comparison Group N = 115. | | |

| **Table B2**  *Independent Sample T-Tests Comparing Baseline-Levels of Indicators for Health, Wellbeing and Life Attitudes Between Complete and Incomplete Twinning Project Responses* | | | | | | | | |
| --- | --- | --- | --- | --- | --- | --- | --- | --- |
|  | Complete | |  | Incomplete | |  |  |  |
| Outcome variable | M | SD |  | M | SD | *t*(df) | *p* | Cohen’s *d* |
| **Health** |  |  |  |  |  |  |  |  |
| Physical health | 3.69 | 0.98 |  | 3.52 | 0.93 | 1.51 (293) | .132 | 0.18 |
| Physical activity | 4.57 | 1.92 |  | 4.11 | 1.97 | 2.05 (293) | .041 | 0.24 |
| **Wellbeing** |  |  |  |  |  |  |  |  |
| State anxiety | 2.08 | 2.63 |  | 2.71 | 2.91 | -1.93 (265.30) | .054 | 0.23 |
| State happiness | 7.58 | 2.04 |  | 7.04 | 2.31 | 2.14 (293) | .034 | 0.25 |
| Psychological need satisfaction | 3.89 | 0.71 |  | 3.73 | 0.73 | 1.98 (293) | .049 | 0.23 |
| **Life Attitudes** |  |  |  |  |  |  |  |  |
| Life satisfaction | 6.83 | 2.02 |  | 6.47 | 1.94 | 1.58 (293) | .116 | 0.18 |
| Custodial attitudes | 4.43 | 0.76 |  | 4.19 | 1.00 | 2.32 (238.41) | .021 | 0.28 |
| Self-efficacy | 4.17 | 0.64 |  | 4.08 | 0.76 | 1.15 (290) | .251 | 0.14 |
| Future optimism | 3.76 | 1.06 |  | 3.48 | 1.30 | 2.00 (247.78) | .046 | 0.24 |
| **Social relations** |  |  |  |  |  |  |  |  |
| Relations with POs | 4.00 | 0.82 |  | 3.84 | 1.07 | 1.41 (238.55) | .159 | 0.17 |
| Relations with prisoners | 4.12 | 0.71 |  | 3.95 | 0.96 | 1.66 (293) | .098 | 0.19 |
| Identification with criminals | 1.97 | 1.09 |  | 2.15 | 1.34 | -1.25 (235.41) | .214 | 0.15 |
| Identification with TP | 3.92 | 0.86 |  | 3.85 | 0.78 | 0.69 (287) | .490 | 0.08 |
| Fusion to criminals | 1.69 | 0.97 |  | 1.96 | 1.17 | -2.17 (282) | .031 | 0.26 |
| Fusion to TP | 3.17 | 1.13 |  | 2.56 | 1.14 | 4.45 (284) | <.001 | 0.53 |
| *Note.* Complete N = 164, Incomplete N = 131 | | | | | | | | |

| **Table B3**  Number of Cases Provided by Institution | | |
| --- | --- | --- |
| Institution | number of cases | number of  complete cases |
| HMP Aylesbury | 28 | 6 |
| HMP Birmingham | 15 | 0 |
| HMP Cookham Wood | 6 | 0 |
| HMP Deerbolt | 12 | 11 |
| HMP Durham | 12 | 11 |
| HMP Exeter | 34 | 19 |
| HMP Hewell | 16 | 14 |
| HMP Holme House | 15 | 9 |
| HMP Kirklevington | 30 | 29 |
| HMP Lewes | 3 | 0 |
| HMP Liverpool | 17 | 6 |
| HMP Oakwood | 1 | 0 |
| HMP Risley | 49 | 31 |
| HMP Stocken | 37 | 22 |
| HMP Stoke Heath | 12 | 0 |
| HMP Sudbury | 10 | 6 |
| HMP Wetherby | 10 | 0 |
| HMP Wormwood Scrubs | 9 | 0 |
| Total | 316 | 164 |

| **Table B4**  *Results of Stepwise Logistic Regression Analysis Predicting Case Completeness Based on Prison (Step 1) and Significant Baseline Wellbeing Differences (Step 2)* | | | | | | | | | | | | | |
| --- | --- | --- | --- | --- | --- | --- | --- | --- | --- | --- | --- | --- | --- |
|  | Case completeness | | | | | | | | | | | | |
| Variables | Step 1 | | | | | |  | Step 2 | | | | | |
|  | B | SE | *Wald* | *df* | *p* | Exp(B) |  | B | SE | *Wald* | *df* | *p* | Exp(B) |
| Constant | -1.253 | 0.46 | 7.32 | 1 | .007 | 0.29 |  | -4.16 | 1.59 | 6.86 | 1 | .009 | 0.02 |
| Prison |  |  | 37.28 | 17 | .003 |  |  |  |  | 35.03 | 17 | .006 |  |
| HMP Birmingham | -19.95 | 10377.78 | 0.00 | 1 | .998 | 0.00 |  | -19.47 | 10155.86 | 0.00 | 1 | .998 | 0.00 |
| HMP Cookham Wood | -19.95 | 16408.71 | 0.00 | 1 | .999 | 0.00 |  | -19.58 | 16329.88 | 0.00 | 1 | .999 | 0.00 |
| HMP Deerbolt | 3.65 | 1.14 | 10.21 | 1 | .001 | 38.50 |  | 3.02 | 1.18 | 6.49 | 1 | .011 | 20.44 |
| HMP Durham | 3.65 | 1.14 | 10.21 | 1 | .001 | 38.50 |  | 3.74 | 1.19 | 9.90 | 1 | .002 | 42.11 |
| HMP Exeter | 1.52 | 0.59 | 6.61 | 1 | .010 | 4.58 |  | 2.14 | 0.68 | 9.97 | 1 | .002 | 8.54 |
| HMP Hewell | 3.12 | 0.89 | 12.34 | 1 | <.001 | 22.75 |  | 3.28 | 0.95 | 11.86 | 1 | <.001 | 26.63 |
| HMP Holme House | 22.46 | 13397.66 | 0.00 | 1 | .999 | 5654161949.98 |  | 22.62 | 13132.17 | 0.00 | 1 | .999 | 6675695990.83 |
| HMP Kirklevington | 4.58 | 1.12 | 16.82 | 1 | <.001 | 98.00 |  | 5.00 | 1.16 | 18.48 | 1 | <.001 | 147.71 |
| HMP Lewes | -19.95 | 23205.42 | 0.00 | 1 | .999 | 0.00 |  | -20.63 | 23126.48 | 0.00 | 1 | .999 | 0.00 |
| HMP Liverpool | 1.10 | 0.72 | 2.30 | 1 | .129 | 3.00 |  | 1.39 | 0.79 | 3.08 | 1 | .079 | 4.00 |
| HMP Oakwood | -19.95 | 40192.97 | 0.00 | 1 | 1.000 | 0.00 |  | -18.32 | 40192.97 | 0.00 | 1 | 1.000 | 0.00 |
| HMP Risley | 2.28 | 0.59 | 14.88 | 1 | <.001 | 9.80 |  | 2.64 | 0.64 | 16.87 | 1 | <.001 | 13.97 |
| HMP Stocken | 1.66 | 0.58 | 8.25 | 1 | .004 | 5.25 |  | 1.88 | 0.65 | 8.44 | 1 | .004 | 6.58 |
| HMP Stoke Heath | -19.95 | 13397.66 | 0.00 | 1 | .999 | 0.00 |  | -19.58 | 12956.85 | 0.00 | 1 | .999 | 0.00 |
| HMP Sudbury | 3.04 | 1.18 | 6.71 | 1 | .010 | 21.00 |  | 3.76 | 1.26 | 8.84 | 1 | .003 | 42.99 |
| HMP Wetherby | -19.95 | 12710.13 | 0.00 | 1 | .999 | 0.00 |  | -18.86 | 12548.54 | 0.00 | 1 | .999 | 0.00 |
| HMP Wormwood Scrubs | -19.95 | 13397.66 | 0.00 | 1 | .999 | 0.00 |  | -19.91 | 13193.31 | 0.00 | 1 | .999 | 0.00 |
| Physical activity |  |  |  |  |  |  |  | -0.09 | 0.10 | 0.81 | 1 | .369 | 0.91 |
| State happiness |  |  |  |  |  |  |  | 0.16 | 0.10 | 2.69 | 1 | .101 | 1.18 |
| Future optimism |  |  |  |  |  |  |  | 0.39 | 0.16 | 5.71 | 1 | .017 | 1.47 |
| Fusion to criminals |  |  |  |  |  |  |  | -0.13 | 0.17 | 0.59 | 1 | .444 | 0.88 |
| Fusion to Twinning Project |  |  |  |  |  |  |  | 0.37 | 0.16 | 5.04 | 1 | .025 | 1.44 |
| Psychological resources |  |  |  |  |  |  |  | -0.34 | 0.29 | 1.40 | 1 | .237 | 0.71 |
| Custodial attitudes |  |  |  |  |  |  |  | 0.21 | 0.19 | 1.19 | 1 | .275 | 1.24 |
| Model summary | χ2 (17) = 165.99, p < .001. (Nagelkerke R^2^ = 59.9) | | | | | |  | Step: Δχ2 (7) = 15.23, p = .033. (ΔNagelkerke R^2^ = 3.9)  Model: χ2 (24) = 181.22, p < .001. (Nagelkerke R^2^ = 63.8) | | | | | |
| *Note*. Case completeness (1 = complete, 2 = incomplete), Prison HMP Aylesbury = indicator. | | | | | | | | | | | | | |

APPENDIX C: Full Results of Manuscript Analyses

| **Table C1**  *Results of Pre-Post Tests for Indicators of Wellbeing, Social Relations and Life Attitudes Among Twinning Project Participants* | | | | | | | | |
| --- | --- | --- | --- | --- | --- | --- | --- | --- |
|  | Pre | |  | Post | |  |  |  |
| Outcome variable | M | SD |  | M | SD | *t*(df) | *p* | Cohen’s *d* |
| **Health** |  |  |  |  |  |  |  |  |
| Physical health | 3.69 | 0.98 |  | 3.79 | 0.85 | -1.23 (163) | .111 | 0.10 |
| Physical activity | 4.57 | 1.92 |  | 4.80 | 1.66 | -1.36 (163) | .087 | 0.11 |
| **Wellbeing** |  |  |  |  |  |  |  |  |
| State anxiety | 2.08 | 2.63 |  | 2.10 | 2.41 | -0.10 (163) | .459 | 0.01 |
| State happiness | 7.58 | 2.04 |  | 7.67 | 2.18 | -0.47 (163) | .321 | 0.04 |
| Psychological need satisfaction | 3.83 | 0.71 |  | 4.00 | 0.60 | -2.94 (163) | .002 | 0.23 |
| **Life Attitudes** |  |  |  |  |  |  |  |  |
| Life satisfaction | 6.83 | 2.02 |  | 7.70 | 1.70 | -5.31 (163) | <.001 | 0.42 |
| Custodial attitudes | 4.41 | 0.78 |  | 1.26 | 1.00 | 1.46 (152) | .147 | 0.12 |
| Self-efficacy | 4.17 | 0.65 |  | 4.34 | 0.64 | -2.71 (161) | .004 | 0.21 |
| Future optimism | 3.76 | 1.06 |  | 3.95 | 1.02 | -1.79 (163) | .076 | 0.14 |
| **Social relations** |  |  |  |  |  |  |  |  |
| Relations with POs | 4.00 | 0.82 |  | 3.96 | 1.06 | 0.45 (163) | .327 | 0.04 |
| Relations with prisoners | 4.12 | 0.71 |  | 4.05 | 1.04 | 0.69 (163) | .245 | 0.05 |
| Identification with criminals | 1.96 | 1.11 |  | 1.93 | 1.10 | 0.26 (121) | .397 | 0.02 |
| Identification with TP | 3.93 | 0.88 |  | 4.21 | 0.87 | -2.93 (140) | .002 | 0.25 |
| Fusion to criminals | 1.67 | 0.94 |  | 1.85 | 1.21 | -1.44 (129) | .072 | 0.13 |
| Fusion to TP | 3.18 | 1.12 |  | 3.33 | 1.26 | -1.13 (141) | .130 | 0.10 |


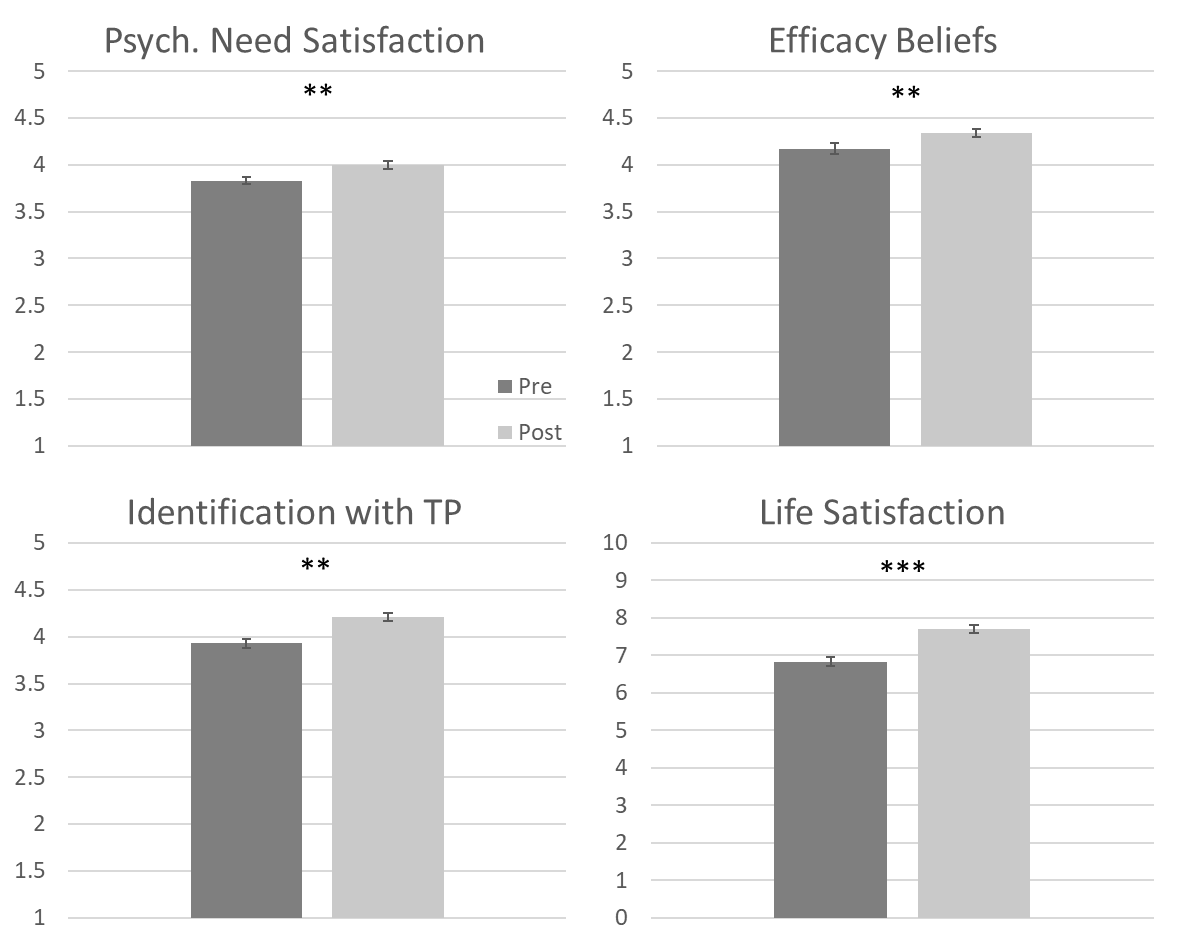


**Figure C1.** *Changes to Wellbeing,* Life A*ttitudes and Identification Among Twinning Project Participants.*

*Note.* Error bars are standard errors based on standard deviations.

| **Table C2**  Correlations and Descriptives Statistics for all Variables Pre-Treatment | | | | | | | | | | | | | | | | | | | | |
| --- | --- | --- | --- | --- | --- | --- | --- | --- | --- | --- | --- | --- | --- | --- | --- | --- | --- | --- | --- | --- |
| Variables |  | M | SD | n | 2. | 3. | 4. | 5. | 6. | 7. | 8. | 9. | 10. | 11. | 12. | 13. | 14. | 15. | 16. | 17. |
| 1. Identification TP |  | 4.20 | 0.87 | 143 | .28*** | -.16* | -.14 | .25** | .26** | .10 | .08 | -.12 | .25** | .16* | .12 | .24** | .18* | -.03 | -.05 | -.09 |
| 1. Fusion TP |  | 3.33 | 1.26 | 144 |  | -.19* | -.04 | .07 | .05 | .04 | .13 | -.10 | .13 | .19* | .08 | .11 | -.04 | .05 | -.17* | -.02 |
| 1. Identification Criminals |  | 1.91 | 1.09 | 128 |  |  | .43*** | -.11 | .00 | -.05 | .00 | .01 | .00 | -.02 | -.06 | .03 | .00 | .13 | -.17 | -.04 |
| 1. Fusion Criminals |  | 1.87 | 1.23 | 137 |  |  |  | -.08 | -.06 | -.09 | -.05 | .23** | -.07 | -.10 | -.16* | -.15 | -.16 | .07 | -.02 | .05 |
| 1. PO relations |  | 3.96 | 1.06 | 164 |  |  |  |  | .63*** | .10 | .15 | -.14 | .12 | .09 | .28*** | .20* | .45*** | -.04 | .03 | -.02 |
| 1. Prisoner relations |  | 4.05 | 1.04 | 164 |  |  |  |  |  | .17* | .14 | -.17* | .31*** | .12 | .35*** | .23** | .57*** | .00 | .00 | .02 |
| 1. Health |  | 3.79 | 0.85 | 164 |  |  |  |  |  |  | .39*** | -.20* | .18* | .24** | .38*** | .28*** | .01 | .02 | -.12 | -.04 |
| 1. Activity |  | 4.80 | 1.66 | 164 |  |  |  |  |  |  |  | -.29*** | .33*** | .36*** | .24** | .37*** | .09 | .20* | -.10 | .04 |
| 1. Anxiety |  | 2.10 | 2.41 | 164 |  |  |  |  |  |  |  |  | -.30*** | -.31*** | -.27*** | -.18* | -.14 | -.17* | .14 | -.17* |
| 1. Happiness |  | 7.67 | 2.18 | 164 |  |  |  |  |  |  |  |  |  | .47*** | .37*** | .66*** | .17* | .25** | -.24** | .07 |
| 1. Psychological need satisfaction |  | 4.00 | 0.60 | 164 |  |  |  |  |  |  |  |  |  |  | .35*** | .51*** | .04 | .50*** | -.12 | .13 |
| 1. Efficacy |  | 4.34 | 0.64 | 163 |  |  |  |  |  |  |  |  |  |  |  | .36*** | .32*** | .09 | -.24** | .26** |
| 1. Life satisfaction |  | 7.70 | 1.70 | 164 |  |  |  |  |  |  |  |  |  |  |  |  | .16* | .20* | -.09 | .08 |
| 1. Custodial Attitudes |  | 4.16 | 1.08 | 164 |  |  |  |  |  |  |  |  |  |  |  |  |  | -.02 | -.01 | .10 |
| 1. Future optimism |  | 3.95 | 1.02 | 164 |  |  |  |  |  |  |  |  |  |  |  |  |  |  | -.26** | .05 |
| 1. Age |  | 31.38 | 7.87 | 139 |  |  |  |  |  |  |  |  |  |  |  |  |  |  |  | -.18* |
| 1. Ethnic minority |  | 0.27 | 0.45 | 164 |  |  |  |  |  |  |  |  |  |  |  |  |  |  |  |  |
| Note. *p < .05, **p < .010, ***p < .001. Ethnic Minority 1 = Yes. | | | | | | | | | | | | | | | | | | | | |

| **Table C3**  *Correlations and Descriptives Statistics for all Variables Post-Treatment* | | | | | | | | | | | | | | | | | | | |
| --- | --- | --- | --- | --- | --- | --- | --- | --- | --- | --- | --- | --- | --- | --- | --- | --- | --- | --- | --- |
| Variables | M | SD | n | 2. | 3. | 4. | 5. | 6. | 7. | 8. | 9. | 10. | 11. | 12. | 13. | 14. | 15. | 16. | 17. |
| 1. Identification TP | 3.92 | 0.86 | 161 | .33*** | -.06 | -.06 | .18* | .02 | .09 | -.05 | -.19* | .23** | .31*** | .18* | .30*** | .01 | .21* | .00 | .08 |
| 1. Fusion TP | 3.17 | 1.13 | 162 |  | -.16 | -.31*** | -.10 | -.13 | .03 | -.01 | -.06 | .05 | .13 | .04 | .15 | -.15 | .15 | -.22** | .18* |
| 1. Identification Criminals | 1.97 | 1.09 | 149 |  |  | .28** | .21* | .20* | -.10 | .03 | .03 | -.10 | -.20* | -.05 | -.10 | .02 | -.23** | .07 | -.11 |
| 1. Fusion Criminals | 1.69 | 0.97 | 156 |  |  |  | .17* | .02 | -.03 | -.05 | -.05 | -.05 | -.12 | -.10 | -.05 | .05 | -.13 | .22* | .08 |
| 1. PO relations | 4.00 | 0.82 | 164 |  |  |  |  | .50*** | .07 | .09 | -.20** | .17* | .18* | .25*** | .15* | .55*** | -.06 | .04 | .02 |
| 1. Prisoner relations | 4.12 | 0.71 | 164 |  |  |  |  |  | .08 | .10 | -.22** | .12 | .25** | .23** | .14 | .58*** | -.03 | -.15 | .07 |
| 1. Health | 3.69 | 0.98 | 164 |  |  |  |  |  |  | .44*** | -.24** | .20** | .17* | .15 | .21** | -.01 | .14 | -.25** | .18* |
| 1. Activity | 4.57 | 1.92 | 164 |  |  |  |  |  |  |  | -.18* | .13 | .06 | .08 | .03 | .01 | -.04 | -.02 | -.01 |
| 1. Anxiety | 2.08 | 2.63 | 164 |  |  |  |  |  |  |  |  | -.36*** | -.36*** | -.12 | -.34*** | -.18* | -.09 | .08 | -.06 |
| 1. Happiness | 7.58 | 2.04 | 164 |  |  |  |  |  |  |  |  |  | .43*** | .04 | .50*** | .04 | .14 | -.19* | .06 |
| 1. Psychological needs | 3.83 | 0.71 | 164 |  |  |  |  |  |  |  |  |  |  | .38*** | .57*** | .07 | .44*** | -.27** | .02 |
| 1. Efficacy | 4.17 | 0.64 | 163 |  |  |  |  |  |  |  |  |  |  |  | .25** | .08 | .13 | -.17* | .10 |
| 1. Life satisfaction | 6.83 | 2.02 | 164 |  |  |  |  |  |  |  |  |  |  |  |  | .07 | .28*** | -.12 | -.05 |
| 1. Custodial Attitudes | 4.43 | 0.76 | 164 |  |  |  |  |  |  |  |  |  |  |  |  |  | -.11 | .07 | .00 |
| 1. Future optimism | 3.76 | 1.06 | 164 |  |  |  |  |  |  |  |  |  |  |  |  |  |  | -.09 | -.01 |
| 1. Age | 31.38 | 7.87 | 139 |  |  |  |  |  |  |  |  |  |  |  |  |  |  |  | -.18* |
| 1. Ethnic Minority | 0.27 | 0.45 | 164 |  |  |  |  |  |  |  |  |  |  |  |  |  |  |  |  |
| *Note.* **p* < .05, ***p* < .010, ****p* < .001. Ethnic Minority 1 = Yes. | | | | | | | | | | | | | | | | | | | |

| **Table C4**  Results of Mediation Analysis Using Process Macro Model 4 (Hayes, 2022) Predicting Change and Post-Treatment Outcomes Based on Identification Change via Psychological Need Satisfaction Change | | | | | | | | | |
| --- | --- | --- | --- | --- | --- | --- | --- | --- | --- |
|  | *Model 1* | | | | | | | | |
|  | Δ Psychological Need Satisfaction | | | |  | Δ Life satisfaction | | | |
|  | B | SE | *95%CI* | *p* |  | B | SE | *95%CI* | *p* |
| *Δ* Psych. Need Satisfaction |  |  |  |  |  | .79 | .23 | .333, 1.237 | <.001 |
| *Δ* Identification with TP | .16 | .06 | .038, .274 | .010 |  | .22 | .16 | -.091, .533 | .164 |
| Age | -.01 | .01 | -.031, .003 | .112 |  | .01 | .02 | -.035, .053 | .691 |
| Disability | .18 | .18 | -.173, .526 | .320 |  | 1.12 | .46 | .216, 2.023 | .016 |
| Ethnic minority | -.29 | .15 | -.592, .018 | .065 |  | -.75 | .40 | -1.548, .042 | .063 |
| Model Summary | F(4,127) = 2.54, p = .043, R2 = .07 | | | |  | F(5,126) = 5.15, p < .001, R2 = .17 | | | |
| Indirect effect |  |  |  |  |  | .12 | .07 | .007, .275 |  |
| Total effect |  |  |  |  |  | .34 | .16 | .026, .659 |  |
|  |  | | | |  |  | | | |
|  | Model 2 | | | | | | | | |
|  | Δ Psychological Need Satisfaction | | | |  | Δ Personal Efficacy | | | |
|  | B | SE | *95%CI* | *p* |  | B | SE | *95%CI* | *p* |
| *Δ* Psych. Need Satisfaction |  |  |  |  |  | .21 | .09 | .036, .391 | .019 |
| *Δ* Identification with TP | .16 | .06 | .038, .275 | .010 |  | .03 | .06 | -.095, .149 | .661 |
| Age | -.01 | .01 | -.031, .004 | .131 |  | .01 | .01 | -.010, .025 | .420 |
| Disability | .18 | .18 | -.172, .532 | .312 |  | -.31 | .18 | -.664, .044 | .856 |
| Ethnic minority | -.28 | .15 | -.587, .026 | .072 |  | -.14 | .16 | -.453, .170 | .369 |
| Model Summary | F(4,125) = 2.46, p = .049, R2 = .07 | | | |  | F(5,124) = 2.54, p = .032, R2 = .09 | | | |
| Indirect effect |  |  |  |  |  | .03 | .02 | .001, .085 |  |
| Total effect |  |  |  |  |  | .07 | .06 | -.061, .182 |  |
|  |  |  |  |  |  |  | | | |
|  | Model 3 | | | | | | | | |
|  | *Δ* Psychological Need Satisfaction | | | |  | Future Optimism (post) | | | |
|  | B | SE | *95%CI* | *p* |  | B | SE | *95%CI* | *p* |
| *Δ* Psych. Need Satisfaction |  |  |  |  |  | .36 | .12 | .124, .602 | .003 |
| *Δ* Identification with TP | .17 | .06 | .063, .285 | .002 |  | .15 | .08 | -.003, .310 | .054 |
| Age | -.02 | .01 | -.039, -.006 | .007 |  | .00 | .01 | -.022, .024 | .913 |
| Disability | .15 | .17 | -.182, .475 | .380 |  | .23 | .23 | -.213, .680 | .302 |
| Ethnic minority | -.27 | .14 | -.558, .014 | .062 |  | -.10 | .20 | -.498, .289 | .599 |
| Future optimism (pre) | -.27 | .06 | -.399, -.147 | <.001 |  | .29 | .09 | .103, .469 | .002 |
| Model Summary | F(5,126) = 5.97, p < .001, R2 = .19 | | | |  | F(6,125) = 4.08, p < .001, R2 = .16 | | | |
| Indirect effect |  |  |  |  |  | .06 | .03 | .013,.138 |  |
| Total effect |  |  |  |  |  | .22 | .08 | .062, .372 |  |
|  |  | | | |  |  | | | |
|  | Model 4 | | | | | | | | |
|  | *Δ* Psychological Need Satisfaction | | | |  | Happiness (post) | | | |
|  | B | SE | *95%CI* | *p* |  | B | SE | *95%CI* | *p* |
| *Δ* Psych. Need Satisfaction |  |  |  |  |  | .66 | .24 | .184, 1.145 | .007 |
| *Δ* Identification with TP | .14 | .06 | .027, .258 | .016 |  | -.03 | .16 | -.356, .290 | .839 |
| Age | -.02 | .01 | -.036, -.002 | .028 |  | -.01 | .02 | -.054, .041 | .782 |
| Disability | .10 | .17 | -.242, .449 | .554 |  | 1.00 | .48 | .058, 1.946 | .038 |
| Ethnic minority | -.26 | .15 | -.558, .039 | .088 |  | -.03 | .42 | -.853, .794 | .943 |
| Happiness (pre) | -.08 | .03 | -.144, -.022 | .008 |  | .40 | .09 | .228, .572 | <.001 |
| Model Summary | F(5,126) = 3.57, p = .005, R2 = .12 | | | |  | F(6,125) = 5.15, p < .001, R2 = .20 | | | |
| Indirect effect |  |  |  |  |  | .09 | .07 | .002, .255 |  |
| Total effect |  |  |  |  |  | .06 | .16 | -.262, .385 |  |
|  |  | | | |  |  | | | |
|  | Model 5 | | | | | | | | |
|  | *Δ* Psychological Need Satisfaction | | | |  | Anxiety (post) | | | |
|  | B | SE | *95%CI* | *p* |  | B | SE | *95%CI* | *p* |
| *Δ* Psych. Need Satisfaction |  |  |  |  |  | -.79 | .26 | -1.307, -.272 | .003 |
| *Δ* Identification with TP | .15 | .06 | .030, .265 | .015 |  | -.09 | .18 | -.439, .268 | .633 |
| Age | -.01 | .01 | -.031, .002 | .080 |  | -.01 | .03 | -.059, .041 | .732 |
| Disability | .17 | .18 | -.178, .517 | .337 |  | -.45 | .52 | -1.469, .578 | .391 |
| Ethnic minority | -.24 | .16 | -.545, .072 | .131 |  | -.47 | .46 | -1.380, .448 | .315 |
| Happiness (pre) | .04 | .03 | -.008, .092 | .097 |  | .26 | .08 | .112, .409 | <.001 |
| Model Summary | F(5,126) = 2.62, p = .027, R2 = .09 | | | |  | F(6,125) = 3.91, p = .001, R2 = .16 | | | |
| Indirect effect |  |  |  |  |  | -.12 | .07 | -.284, -.002 |  |
| Total effect |  |  |  |  |  | -.20 | .18 | -.558, .155 |  |

APPENDIX D: Overview of survey items and scales

| **Measure**/ Items | Scale | (T1) α | (T2)  α |
| --- | --- | --- | --- |
| **Health** |  |  |  |
| In general, would you say your health is… | 1 = Poor, 2 = Fair, 3 = Good, 4 = Very good, 5 = Excellent |  |  |
| **Physical activity** |  |  |  |
| In the past week, on how many days have you done a total of 30 minutes or more of physical activity, which was enough to raise your breathing rate? | 1 – 7 |  |  |
| **Anxiety** | 0 – 10 |  |  |
| On a scale of 0-10, where 0 is not at all anxious and 10 is completely anxious, overall, how anxious did you feel yesterday? |  |  |  |
| **Happiness** |  |  |  |
| On a scale of 0-10, where 0 is not at all happy and 10 is completely happy, overall, how happy did you feel yesterday? | 0 – 10 |  |  |
| **Psychological need satisfaction** |  | .87 | .84 |
| “Please select the answer that best describes your experience over the last 2 weeks:…”  I've been dealing with problems well | 1 = None of the time, 2 = Rarely, 3 = Some of the time, 4 = Often, 5 = All of the time |  |  |
| I've been feeling useful | “ |  |  |
| I've been feeling relaxed | “ |  |  |
| I've been thinking clearly | “ |  |  |
| I've been feeling close to other people | “ |  |  |
| I've been able to make up my own mind about things | “ |  |  |
| **Life satisfaction** |  | .76 | .73 |
| On a scale of 0-10, where 0 is not at all worthwhile and 10 is completely worthwhile, overall, to what extent do you feel the things you do in your life are worthwhile? | 0 – 10 |  |  |
| On a scale of 0-10, where 0 is not at all satisfied and 10 is completely satisfied, overall, how satisfied are you with your life nowadays? | 0 – 10 |  |  |
| **Custodial attitudes** |  | .88 | .94 |
| I am motivated to work towards goals and targets | 1 = Strongly Disagree, 2 = Disagree, 3 = Neither agree nor disagree, 4 = Agree, 5 = Strongly agree |  |  |
| I use my time in this prison as a chance to change | “ |  |  |
| I am motivated to address my offending behaviour | “ |  |  |
| **Self-efficacy** |  |  |  |
| ‘I can achieve most of the goals I set myself‘? | 1 = Strongly Disagree, 2 = Disagree, 3 = Neither agree nor disagree, 4 = Agree, 5 = Strongly agree |  |  |
| **Future optimism** |  |  |  |
| I've been feeling optimistic about the future | 1 = Strongly Disagree, 2 = Disagree, 3 = Neither agree nor disagree, 4 = Agree, 5 = Strongly agree |  |  |
| **Relations with POs** |  |  |  |
| Personally, I get on well with the officers on my wing | 1 = Strongly Disagree, 2 = Disagree, 3 = Neither agree nor disagree, 4 = Agree, 5 = Strongly agree |  |  |
| **Relations with prisoners** |  |  |  |
| My relationships with other prisoners in this prison are good | 1 = Strongly Disagree, 2 = Disagree, 3 = Neither agree nor disagree, 4 = Agree, 5 = Strongly agree |  |  |
| **Identification with criminals** |  |  |  |
| "I identify with a criminal group (in or outside prison, e.g. a gang)" | 1 = Strongly Disagree, 2 = Disagree, 3 = Neither agree nor disagree, 4 = Agree, 5 = Strongly agree |  |  |
| **Identification with TP** |  |  |  |
| "I identify with the Twinning project" | 1 = Strongly Disagree, 2 = Disagree, 3 = Neither agree nor disagree, 4 = Agree, 5 = Strongly agree |  |  |
| **Fusion to criminals** |  |  |  |
| Imagine the small circle is YOU and the big circle is a criminal group (in or outside prison, such as a gang or friends who deal drugs). Which option above best represents your relationship to a criminal group?  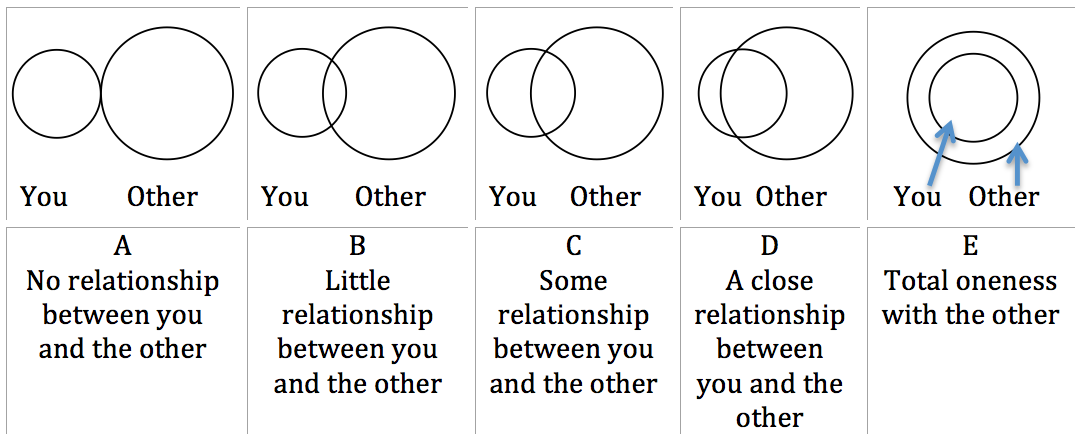 | A - No relationship between you and the other, B - Little relationship between you and the other, C - Some relationship between you and the other, D - A close relationship between you and the other, E - Total oneness with the other |  |  |
| **Fusion to TP** |  |  |  |
| Imagine the small circle is YOU and the big circle is the Twinning Project. Which option above best represents your relationship to the Twinning Project? 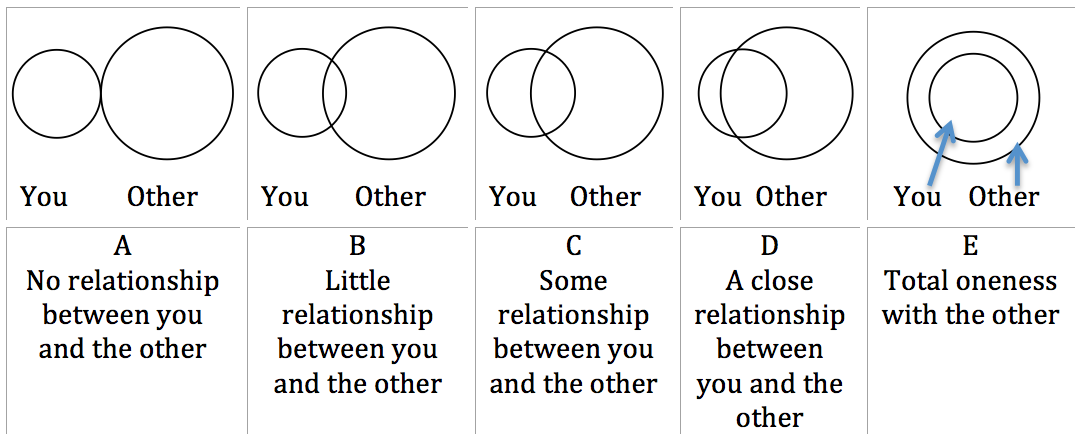 | A - No relationship between you and the other, B - Little relationship between you and the other, C - Some relationship between you and the other, D - A close relationship between you and the other, E - Total oneness with the other |  |  |
